# The modulation of emotional and social behaviors by oxytocin signaling in limbic network

**DOI:** 10.3389/fnmol.2022.1002846

**Published:** 2022-11-17

**Authors:** Rodrigo Triana-Del Rio, Sayali Ranade, Jahel Guardado, Joseph LeDoux, Eric Klann, Prerana Shrestha

**Affiliations:** ^1^Center for Neural Science, New York University, New York, NY, United States; ^2^Department of Neurobiology and Behavior, School of Medicine, Stony Brook University, Stony Brook, NY, United States

**Keywords:** oxytocin, oxytocin receptor (OTR), intracellular cascades, social behavior, emotional behavior, threat response, stress response

## Abstract

Neuropeptides can exert volume modulation in neuronal networks, which account for a well-calibrated and fine-tuned regulation that depends on the sensory and behavioral contexts. For example, oxytocin (OT) and oxytocin receptor (OTR) trigger a signaling pattern encompassing intracellular cascades, synaptic plasticity, gene expression, and network regulation, that together function to increase the signal-to-noise ratio for sensory-dependent stress/threat and social responses. Activation of OTRs in emotional circuits within the limbic forebrain is necessary to acquire stress/threat responses. When emotional memories are retrieved, OTR-expressing cells act as gatekeepers of the threat response choice/discrimination. OT signaling has also been implicated in modulating social-exposure elicited responses in the neural circuits within the limbic forebrain. In this review, we describe the cellular and molecular mechanisms that underlie the neuromodulation by OT, and how OT signaling in specific neural circuits and cell populations mediate stress/threat and social behaviors. OT and downstream signaling cascades are heavily implicated in neuropsychiatric disorders characterized by emotional and social dysregulation. Thus, a mechanistic understanding of downstream cellular effects of OT in relevant cell types and neural circuits can help design effective intervention techniques for a variety of neuropsychiatric disorders.

## Introduction

**FIGURE 1:**
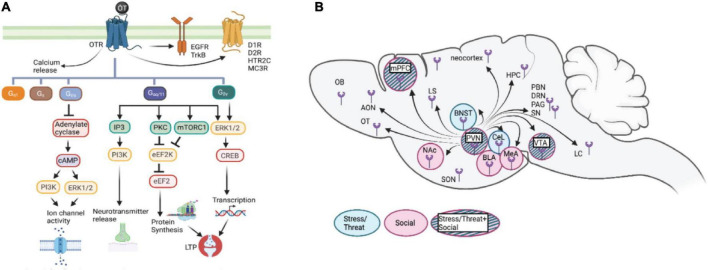
**(A)** Schematic representation of potential cascades involved in oxytocin signaling: OTR which is G-protein coupled receptor acts through various G-protein subunits, Gaq111 being the most common. This subunit either through calcium release or EGFR/TrkB transactivation brings about various downstream effects including neurotransmitter release, protein synthesis. Additionally binding of OT to its receptor also trans-activates some other receptors like dopamine receptors (01R, D2R), serotonin receptor (5HTR2c), orexin receptor (OXR) and melanocortin receptor (MC3R) forming heterodimers. Other subunits include Gpy; involved in LTP induction and protein synthesis, Gifo in regulating ion channel activity, Gs and Ga1 involved in cellular responses like proliferation and differentiation. **(B)** Diagram of oxytocin release in brain areas involved in stress/threat or social networks. Oxytocin stimulates the limbic forebrain through the presence of OTR and intracellular cascades, increasing the signal to noise ratio for stress/threat or social information. OT, oxytocin; OTR, oxytocin receptor; Gaq111, Gv0, Gs, Ga1; Gifo various G-protein subunits, EGFR, Epidermal growth factor; cAMP, cyclic adenosine monophosphate; IP3, Inositol 1,4,5 triphosphate; PKA, protein kinase A; pKC, Protein Kinase C; ERK, extracellular signal related kinases; eEF2, elongation factor 2; CREB, Cyclic AMP responsive element binding protein; LTP, Long term potentiation; 01R, Dopamine type1 receptor; D2R, Dopamine type 2 receptor; HTR2C, 5Hydroxytryptamine receptor 2C; MC3R, Melanocortin 3 receptor; OXR, Orexin receptor; ERK1/2, Extracellular signal-regulated kinase; mTORC1, mammalian target of rapamycin complex I; PVN, paraventricular nucleus; SON, supraoptic nucleus; mPFC, medial prefrontal cortex; OB, olfactory bulb; OT, olfactory tubercle; AON, anterior olfactory nucleus; BNST, base nucleus of the stria terminalis; CeL, centrolateral amygdala; BLA, basolateral amygdala; MeA, medial amygdala; Nae, nucleus accumbens; VTA, ventrotegmental nucleus; LS, lateral septum; HPC, hippocampus; LC, locus coeruleus; PAG, periaqueductal gray; SN, substantia nigra; PBN, parabrachial nucleus; ORN, dorsal raphe nucleus.

Well-known for its role in reproductive behaviors, the highly conserved neuropeptide oxytocin (OT) is a key modulator of neural activity in a distributed network of cells within the limbic forebrain. OT modulation of the limbic forebrain network is critical for mediating behavioral and physiological responses to internal and external stimuli – prominent among these are aversive stress/threat and appetitive social exposure. A wide range of behaviors modulated by OT include the threat and/or stress-induced defensive responses such as freezing and pro-active coping, and social exposure-elicited behaviors such as social interaction, aggression, mating, and maternal care (Carter, 1998; Ferguson et al., 2000; Mantella et al., 2003; Pedersen et al., 2006; Neumann, 2008; Bartz et al., 2011). OT modulation is important for conferring salience of emotional and social behaviors (Carter, 1998; Ferguson et al., 2000; Ciocchi et al., 2010; Bartz et al., 2011; Knobloch et al., 2012; Hasan et al., 2019). The pleiotropic effects of OT on these behaviors are thought to be driven by cascading molecular signaling in specific neural circuits across the brain (Figure 1).

Oxytocin (OT) binds almost exclusively to its membrane-bound oxytocin receptor (OTR), which is a G-protein coupled receptor that can associate with one of several G proteins that mediate distinct physiological responses downstream of OT-OTR interaction in a cellular context and circuit-specific manner (Tomizawa et al., 2003; Wang and Hatton, 2007; Lin et al., 2012; Chini et al., 2017). The knowledge of various signaling pathways underlying context and experience-dependent behavioral responses induced by OT is crucial for understanding the heterogeneity of the downstream effects elicited by OT across the limbic forebrain. The critical challenge in oxytocin research is establishing causal relations between the specific behavioral effect and the underlying molecular mechanisms and circuits. The objective of this review is to provide a framework for the role that OT plays in modulating physiological responses relevant to emotional and social behaviors at different levels of the organization – from signaling inside cells to specific cell types and neural circuits, based on rodent studies. We describe here the cellular and molecular mechanisms that underlie the neuromodulation by oxytocin and how OT signaling in specific neural circuits within the limbic forebrain mediates emotional and social behaviors. In light of the array of modulatory effects that OT exerts on complex behaviors in humans and animal models, we discuss how OT signaling has been a prime focus for understanding pathophysiology and therapeutic potential in neuropsychiatric disorders.

## Oxytocin signaling inside cells

In mammals, OT is synthesized in the hypothalamus, particularly in the paraventricular nucleus (PVN), the supraoptic nucleus (SON), and accessory magnocellular nuclei (AN) (Stoop, 2012). OT is released from these nuclei as a neurohormone with both paracrine and synaptic functions; therefore, oxytocin release has a dual mechanism, reaching different cortical and subcortical regions where the oxytocin receptor (OTR) is expressed (Jiménez et al., 2015; Mitre et al., 2016; Jurek and Neumann, 2018). The release of OT from PVN and SON, *via* axonic fibers, stimulates numerous brain regions expressing the OTR (Knobloch et al., 2012; Mitre et al., 2016) and permits the precise time-dependent, local regulation of its basal levels in addition to volume transmission (Landgraf and Neumann, 2004). Aside from axonal release, the calcium-dependent release of OT from dendrites is triggered by vesicles primed for activity-dependent release, which could lead to a functional reorganization of neuronal networks harboring the OTR, thereby affording a substrate for sustained effects (Ludwig et al., 2002).

### Oxytocin binds to its cognate receptor oxytocin receptor

The various effects exerted by OT are channeled through its only receptor, the oxytocin receptor (OTR), which belongs to the G-protein coupled receptor family (Figure 1A). In rare circumstances, OT can also bind to the receptor of closely related nonapeptide vasopressin, albeit with lower affinity (Wang and Hatton, 2007). The OTR is expressed in various cell populations within a distributed network across the brain and binds OT with an affinity of about 1–10 nM (Chini et al., 2017). This cell surface receptor transmits the OT signal intracellularly, enabling it to exert its effect on various cell functions. There are several well-established pathways downstream of OT-OTR interactions, which vary at the level of brain regions, cells, and molecules involved in other outcomes of these interactions. Context-dependent signaling is central to the variability of OTR activity, hence it is important to understand how cellular context influences OT-mediated activation of various intracellular signaling cascades and the recruitment of additional signaling partners. Along the temporal scale, the physiological effects exerted by oxytocin are both immediate and/or long-lasting. The immediate effects of oxytocin can be attributed to the specific signaling cascade it activates, leading to biochemical changes. The enduring effects exerted by OT, on the other hand, are due to its relatively long half-life and the cascading intracellular messenger pathways that lead to altered gene expression (Ludwig and Leng, 2006). Additionally, OT can transactivate members of the receptor tyrosine kinase family – TrkB, which is a receptor for brain-derived neurotrophic factor (BDNF) (Mitre et al., 2022) and epidermal growth factor receptor (EGFR) (Lin et al., 2012). OT-mediated TrkB transactivation leads to clustering of the scaffold protein gephyrin and mediates inhibitory responses. Moreover, *in vitro* and *in vivo* data suggest that G protein-coupled receptors, including OTR, can form heterodimers with other multiple G protein-coupled receptors, including dopamine D1 and D2, serotonin 2C, orexin, and melanocortin 3 receptors (MCR3), providing possible mechanisms for its many physiological effects (Ringuet et al., 2021).

### Oxytocin and G protein signaling

Oxytocin receptor (OTR), a G-protein coupled receptor, binds to downstream coupling partners referred to as G proteins that contain three subunits – α, β and γ (Kamato et al., 2015). While all three subunits are crucial, the specificity of the downstream signaling is generally conferred by the engaged α subunit, which can be Gα_*q*_, Gα_*s*_, Gα_*i*_, and others. OTRs have been shown to couple to Gα_*q*_ and Gα_*i/o*_ subtypes of G proteins, which have contrasting effects on cellular activity and cAMP abundance. An important factor in determining the G protein coupling partner of OTR is the oxytocin concentration. At a low concentration of OT (∼ 2 nM), Gα_*q*_ is the partner of choice, whereas, at a high OT concentration of ∼90 nM, OTR is coupled to Gα_*oB*_. The coupling partners for the intermediate concentrations of OT (11 nM – 62 nM) include Gα_*i*3_, Gα_*oA*_, Gα_*i*1_, and Gα_*i*2_ (Busnelli et al., 2013).

Of physiological relevance, OTR couples predominantly to Gα_*q/*11_ subunit. Downstream of Gα_*q/*11_ coupling, OTR stimulates intracellular calcium (Ca^2+^) mobilization through a phospholipase C (PLC)-dependent mechanism (Park et al., 1998; Gutkowska and Jankowski, 2012). PLC stimulates hydrolysis of the phospholipid phosphatidylinositol 4,5-bisphosphate (PIP_2_) to diacylglycerol (DAG), which in turn activates protein kinase C (PKC) and inositol 1,4,5-trisphosphate (IP_3_). This cascade of molecular signaling stimulates the release of intracellular Ca^2+^ stores *via* IP_3_ receptors and also activates other Ca^2+^-activated kinases (Thibonnier et al., 1999; Viero et al., 2010). However, there is a lot of heterogeneity at the level of the physiological outcome of OT-OTR activation. For example, in neuronal cells – an increase in Ca^2+^ concentration leads to the formation of Ca^2+^-calmodulin complexes which then activate neuronal isoforms of nitric oxide (NO) synthase. NO in turn stimulates the soluble guanylate cyclase to produce cGMP. In neurosecretory cells, rising Ca^2+^ levels regulate their firing pattern, thereby modulating cellular excitability and leading to transmitter release (Gimpl and Fahrenholz, 2001). Further, elevated Ca^2+^- levels can induce alterations in gene expression, both at the levels of transcription and protein synthesis (translation). G_β γ_ subunits coupled to OTR are predominantly involved in the peripheral actions of the neuropeptide (Hoare et al., 1999; Zhong et al., 2003). In neurons, however, these subunits are implicated in modulating electrical activity including dissociated hippocampal neurons (Blumenstein et al., 2004) as well as in the brainstem and spinal cord (Yevenes et al., 2003).

Oxytocin receptor (OTR) coupling to different G proteins mediates diverse effects depending on the cellular context. Whole-cell patch-clamp recordings from the supraoptic nucleus in brain slices have demonstrated that OT induces Gα_*q/*11_-mediated calcium mobilization which is followed by subsequent spike frequency reduction in response to the progressive elevation in OT concentration (Wang and Hatton, 2007). Similarly, in the midbrain, OT directly increases the firing rate of dopamine neurons in the ventral tegmental area (VTA), which is consistent with the concept that OTR is primarily coupled to Gα_*q/*11_ protein (Xiao et al., 2017). Primary cerebellar neurons display OT-mediated modulation in the levels of neurexin and neuregulin. This involves the regulation of Rho GTPases and OTR coupling with Gα_*q/*11_ (Zatkova et al., 2016). Finally, In the GN11 cell line which is derived from olfactory neurons, OTR coupling to Gα_*q*_ has been shown to decrease inward rectifying potassium K + (IRK) currents, however, in contrast, the opposite effect of increasing K + involves OTR coupling to Gα_*i/o*_ in a different sub-population (Gravati et al., 2010). Thus, intracellular OTR coupling to specific G proteins determines the cellular response and activity to OT-OTR binding.

### Oxytocin and synapse dynamics

The effect of OT on synaptic activity and synapse formation has been well established (Bakos et al., 2018). Neurodevelopmental disorders, including autism spectrum disorders, have various synaptopathies that present with deficient oxytocin signaling (Bakos et al., 2015). The multiple properties of synapses modulated by OT include synapse formation (Blumenstein et al., 2004), synapse stabilization (Lestanova et al., 2016), synapse number (Ripamonti et al., 2017), and synaptic transmission (Jo et al., 1998). Disrupted synaptogenesis and synapse functionality can result in impaired neuronal connectivity, circuit formation, and stability (Zatkova et al., 2016). OT modulates synaptic features by acting on local synaptic sites and induces context-dependent signaling cascades. At the presynaptic membrane, activation of OTR leads to increased intracellular Ca^2+^ concentration, which may result in increased neurotransmitter release (Bakos et al., 2018). This increase in Ca^2+^ could be due to G_α *q*_-mediated activation of the phospholipase-C leading to inositol 1,4,5-trisphosphate receptor (IP3R) induced calcium release from intracellular sources (Lambert et al., 1994) and through inhibition of the potassium Kir7.1 channels, which induces calcium entry through the voltage-dependent calcium channel (York et al., 2017).

### Oxytocin modulates long-term synaptic plasticity and protein synthesis

Oxytocin exerts its effects on emotional behaviors by recruiting converging pathways in the paraventricular nucleus that promote nascent protein synthesis (Blume et al., 2008; Knobloch et al., 2012), innervating for example the central amygdala, where OTR-expressing neurons express higher synaptic strength after proactive coping threat conditioning and freezing reduction (Terburg et al., 2018). Protein synthesis is tightly regulated at the level of initiation and elongation (Shrestha and Klann, 2022). OT promotes translation elongation by activating eukaryotic elongation factor 2 (eEF2) both in a hypothalamic cell line and *in vivo* within the PVN (Martinetz et al., 2019). Protein synthesis is required for long-term synaptic plasticity, which is a persistent change in the synaptic strength, manifested as either long-term potentiation (LTP) or long-term depression (LTD). OT has been shown to affect both forms of synaptic plasticity depending on the brain region. In the medial amygdala (MeA), OT strongly augments LTD induction in the afferents from the accessory olfactory bulb (AOB) (Gur et al., 2014). In the rat hippocampus, OT is involved in the maintenance of LTP, which is induced upon tetanic stimulation in the CA1 region. This effect of oxytocin is exerted via two pathways – the first pathway involves the conventional Gα_*q/*11_-coupled phospholipase C pathway, whereas the second pathway involves transactivated EGFR (Wang, 2016) downstream signaling mediated by phosphatidylinositol 3 kinase (PI3K), extracellular signal-regulated kinase 1/2 (ERK1/2) and mammalian target of Rapamycin complex I (mTORC1) (Tomizawa et al., 2003; Lin et al., 2012). Importantly OTR-mediated mediated enhancement of LTP is dependent on new protein synthesis (Lin et al., 2012), indicating that OT triggers long-lasting effects on cellular physiology – which is particularly relevant for long-term social and threat-related memories.

## Limbic forebrain-based oxytocin signaling network in stress/threat responses

Oxytocin-oxytocin receptor (OT-OTR) interactions mediate neuromodulatory influence on specific neural motifs within the emotional limbic forebrain for the acquisition and expression of stress/threat responses (Figure 1B). OT modulation serves the important function of fine-tuning the salience of neuronal network activity, mainly facilitating GABA-dependent increase in signal-to-noise ratio (Owen et al., 2013; Mitre et al., 2016; Oettl et al., 2016; Tirko et al., 2018), although its role in glutamatergic and astrocyte modulation has been addressed recently (Tirko et al., 2018; Wahis et al., 2021). This can be demonstrated at the level of behavior using a differential threat conditioning paradigm in which animals are exposed to two different auditory stimuli, one that is associated with the imminence of a footshock (i.e., threat) and the other that predicts safety. Pavlovian threat conditioning generates cued freezing response to threat predictive stimulus whereas instrumental signaled active avoidance results in cued avoidance responses that dominate over freezing (LeDoux and Daw, 2018; Hasan et al., 2019). OT neuromodulation of pertinent neural circuits facilitates the discrimination between stimuli that predict threat and safety, respectively. Supported by human studies, OT has been shown to facilitate adaptive response to threats and to reduce maladaptive fear, notably in social contexts (Janeček and Dabrowska, 2019; Triana-Del Río et al., 2019; van den Burg and Hegoburu, 2020), and mainly through its OT releasing long-range axons that irrigate the limbic forebrain (Sofroniew, 1983; Knobloch et al., 2012). Although acute activation of OTR in the central nervous system promotes the attenuation of paralyzing threat responses (freezing), high doses of OT administered chronically elicit anxiety-like responses (Peters et al., 2014). This dose-dependent effect may be mediated by OT binding to arginine vasopressin (AVP) receptors, which have been associated with enhanced fear (Viviani et al., 2011; Jurek and Neumann, 2018).

Oxytocin (OT) signaling is also critical for the physiological and behavioral responses to different kinds of stressors, which can be broadly categorized into physical and psychological stressors. Physical stress represents potentially life-threatening bodily harm, such as electric footshock and forced swim stressors whereas psychological stress involves anticipation of pain or threat, for instance - maternal separation, immobilization, social defeat, and predation (Sandi and Haller, 2015). Depending on the context and the stressor type, OT either impedes the already operating stress response or is released simultaneously with the onset of a stressor to moderate the outcome of the stress response (Winter et al., 2021). OT signaling in brain regions within the limbic forebrain network, including the paraventricular thalamus, amygdala, prefrontal cortex, lateral septum, and bed nucleus of stria terminalis, orchestrate the neuromodulation of stress/threat responses (Table 1).

**TABLE 1:** OT-OTR signaling in limbic network modulates neuronal activity and behavioral salience in circuits for stress/threat and social responses.

Component of limbic forebrain network	Role in stress/threat responses	Neuromodulation by OT-OTR
Paraventricular Nucleus of the Hypothalamus	Triggers and modulates stress responses through the production of Corticotropin releasing factor (CRF), Adrenocorticotropic hormone (ACTH or CORT), Oxytocin (OT) and Vasopressin (AVP). First relay of the hypothalamus-pituitary-adrenal axis (HPA) (Marques de Souza and Franci, 2008; Dabrowska et al., 2011; Cox et al., 2015; Gómez-Gómez et al., 2019)	OT has anti-stress and anxiolytic effects, by the inhibition of CRF & CORT production. OT release also responds to conditioned threat stimuli (Arima and Aguilera, 2000; Liu et al., 2016; Grund et al., 2017; Hasan et al., 2019; Duarte-Guterman et al., 2020)
Centrolateral Amygdala	Triggers and modulates conditioned threat responses, specially freezing in rodents (Ciocchi et al., 2010; Haubensak et al., 2010; Duvarci et al., 2011; Fadok et al., 2017; Shrestha et al., 2020b)	OT stimulates the OTR-expressing PKCd cells that inhibit the AVPr-expressing SOM cells, thus inhibiting the display of freezing behavior (Huber et al., 2005; Kirsch et al., 2005; Knobloch et al., 2012; Terburg et al., 2018; Ferretti et al., 2019; Gunduz-Cinar et al., 2020)
Prefrontal Cortex	Cognitive control of stress/threat responses through top-down modulation of subcortical areas, to initiate behavior and decision-making (Milad and Quirk, 2002; Gruene et al., 2015; Capuzzo and Floresco, 2020; Diehl et al., 2020; Scheggia et al., 2020)	OT reduces stress/threat responses (freezing) through OTR-expressing GABAergic cells. OT stimulates social buffering of freezing responses (Nakajima et al., 2014; Sabihi et al., 2014; Li et al., 2016; Jang et al., 2022)
Bed nucleus of stria terminalis	Integrates information by interfacing with other brain regions to regulate distinct aspects of motivated behaviors associated with stress, anxiety, depression, and decision-making (Janeček and Dabrowska, 2019; Mosley et al., 2021)	OT through OTR facilitates cued freezing but reduces freezing responses to un-signaled, diffuse threats, underlying discrimination among the type of threat (Martinon and Dabrowska, 2018; Francesconi et al., 2021)
**Component of limbic forebrain network**	**Role in social responses**	**Neuromodulation by OT-OTR**
Paraventricular Nucleus of the Hypothalamus	Increases arousal for relevant stimuli through neuroendocrine regulation (Smith and Wang, 2014; Peñagarikano et al., 2015)	Neuronal activity in OT neurons is positively correlated to social interaction (Smith and Wang, 2014; Peñagarikano et al., 2015)
Medial Amygdala	Increases behavioral salience for both positive and negative emotional stimuli (Arakawa et al., 2010; Senn et al., 2014)	Neuronal activity and synaptic plasticity in OTR-expressing neurons of the medial amygdala are positively correlated to social interaction (Choleris et al., 2007; Lukas et al., 2013; Gur et al., 2014)
Prefrontal cortex	Modulates social behavior and relays salience information to other brain areas (Jang et al., 2022; Musardo et al., 2022)	Neuronal activity from projections to and from the mPFC can be modulated by OT (Tan et al., 2019; Musardo et al., 2022).
Mesolimbic dopaminergic system	Integrates information for reward, reinforcement and motivated behaviors (Dölen, 2015; Wei et al., 2015)	OT stimulates OTR expressing cells in the ventro tegmental area and nucleus accumbens to increase reward salience for social stimuli, thus increasing social interaction (Hung et al., 2017; Peris et al., 2017; Kohli et al., 2019; Nardou et al., 2019)

### Oxytocin is released by paraventricular nucleus during stress/threat responses

Oxytocin (OT) is released from the PVN during or immediately after, acute stress. Available rodent models of stress partially mimic the stress-induced pathophysiological and behavioral changes as seen in humans. In the acute model, the stressor is applied once and for a short time, while chronic stress involves repeated application of stressful stimuli over an extended period (Daviu et al., 2019). OT modulation of the stress response is particularly evident during periods of high endogenous OT levels such as the peripartum period (Slattery and Neumann, 2008). During this period, OT reduces the levels of corticotropin-releasing factor (CRF) in the PVN and the sympathetic nervous system as a physiological response to labor-induced stress. CRF release in PVN is the primary inducer of the hypothalamic-pituitary-adrenal (HPA) axis that activates generalized stress responses. For instance, in lactating women, the increase in OT during lactation appears to dampen the subsequent stress-induced secretion of corticosteroid (CORT) (Cox et al., 2015). The bidirectional inflection of OT and CRF in the PVN could underlie one of the OT-dependent neural mechanisms of stress-buffering. The cellular architecture of PVN comprises magnocellular and parvocellular neurosecretory cells, that have distinct morphology, electrophysiological properties, and pathways. While the magnocellular PVN cells innervate numerous forebrain regions and release neurohormones into the blood from the posterior pituitary, parvocellular neurosecretory cells in PVN regulate the anterior pituitary via projections to the medial eminence. Evidence for a direct interaction between the OT and CRF systems is based on the observation that parvocellular CRF neurons in the PVN co-express OT but not OTR, whereas magnocellular CRF neurons co-express CRF receptor (CRFR2), OT, and OTR, allowing these CRF neurons to respond to OT release and vice versa (Arima and Aguilera, 2000; Dabrowska et al., 2011).

Furthermore, chronic administration of OT modulates the expression of the *crfr2* gene, leading to a reduction in CRFR2 membrane expression and eventually an anxiogenic phenotype (Winter et al., 2021). Additional anxiolytic mechanisms in the PVN related to OT include the stimulation of oxytocin secretion via GABA-B receptors (Marques de Souza and Franci, 2008) and activation of OT neurons via secretion of neuropeptide-S (Grund et al., 2017). Oxytocin also increases the pain threshold and stimulates various positive social interactions (Rahm et al., 2002; Uvnäs Moberg et al., 2020). During the acquisition of cued freeze suppression and/or extinction in threat conditioning paradigms, coordinated release of OT and glutamate from irrigated areas innervated by their axonal terminals to induce network plasticity in pertinent brain regions including the central amygdala, prefrontal cortex, lateral septum, and bed nucleus of the stria terminalis (BNST) (Knobloch et al., 2012; Hasan et al., 2019). Recent studies strongly suggest that the connectivity of the PVN to these areas involves experience-dependent control of spiking activity in the PVN, with hypothalamic OT neurons constituting a memory engram for the extinction of learned freezing responses (Hasan et al., 2019). Additionally, repeated exposure to OT produces long-lasting effects by affecting the activity of other transmission systems like dopamine, a mechanism that makes OT potentially clinically relevant. Interestingly, its function to modulate threat responses depends on the context and internal states. OT signals can either reduce or exacerbate such reactions, depending on the context, social environment, and hormonal status (Bosch and Neumann, 2012).

### Oxytocin modulates stress/threat responses in the amygdala

The centrolateral nucleus of the amygdala (CeL) is implicated in the acquisition, storage, expression, and extinction of threat-dependent memories (Ciocchi et al., 2010; Haubensak et al., 2010; Duvarci et al., 2011; Fadok et al., 2017; Terburg et al., 2018; Winter et al., 2021) and the ensuing physiological response of retrieving those memories: freezing behavior (Ciocchi et al., 2010; Viviani et al., 2011; Whittle et al., 2021) or adaptive signaled avoidance (Fadok et al., 2017; Terburg et al., 2018; van den Burg and Hegoburu, 2020). Two prominent neuronal populations in the CeL differ in their reactivity to threat-predictive conditioned stimuli (CS): one population exhibits excitatory (CeL-On) while the other population exhibits inhibitory (CeL-Off) responses to the CS following Pavlovian threat conditioning (Ciocchi et al., 2010; Haubensak et al., 2010; Duvarci et al., 2011). Moreover, these cell types have been genetically identified and manipulated using cre- driver lines in rodents: CeL-On cells have been shown to express somatostatin (Som +) (Li et al., 2013; Penzo et al., 2015), and CeL-Off cells correspond to PKCδ + neurons, which in turn express the oxytocin receptor (Haubensak et al., 2010; Shrestha et al., 2020b). Further research has shown that, under baseline conditions, CeL-Off (PKCδ +) neurons exert a tonic inhibitory influence on cells within the centromedial nucleus (CeM), the output nucleus of the amygdala. Excitation of CeL-On (Som +) cells during CS would cause inhibition of CeL-Off neurons, resulting in disinhibition of freezing output neurons within CeM (Ciocchi et al., 2010; Haubensak et al., 2010; Fadok et al., 2017). Therefore, both CeL-On and CeL-Off neurons project to the CeM and inhibit each other (Ciocchi et al., 2010; Haubensak et al., 2010). However, an alternative view indicates a higher level of complexity for this network, in which connections between like neurons are stronger than those between different neuronal types (Hunt et al., 2017). Both CeL-Off (Som +) and CeL-On (PKCδ +) synapses are enhanced after Pavlovian threat conditioning for the expression of either freezing or active avoidance responses, respectively (Li et al., 2013; Penzo et al., 2014; Terburg et al., 2018). Therefore, these cell populations interact in a network with mutual inhibition and differentially encode memory acquisition with threat and safety cues by altering the cellular translation landscape (Shrestha et al., 2020b). In this study, blocking *de novo* protein synthesis in CeL-PKCδ interneurons disrupts the acquisition and consolidation of long-term inhibition of the conditioned freezing response and threat/safety discrimination (Shrestha et al., 2020b,a). Conversely, the inactivation of Som + neurons impedes the acquisition of freezing responses, while their optogenetic activation stimulates them. This also suggests that threat conditioning may disrupt competition between mutually inhibitory CeL neuron subtypes (Li et al., 2013; Penzo et al., 2014).

While OT can act on different subnuclei of the amygdala to induce freezing extinction/suppression (Gunduz-Cinar et al., 2020), the well-described mechanism of action has been done in the CeL – where PKCδ + cells are stimulated by OT and glutamate from the PVN and lateral amygdala (LA), respectively (Huber et al., 2005; Viviani et al., 2011; Terburg et al., 2018; Hasan et al., 2019). The coordinated release of OT and glutamate in CeL activates CeL-Off (PKCδ +) cells and subsequently inhibits CeL-On (Som +) cells, leading to the attenuation of conditioned freezing (Knobloch et al., 2012; Hasan et al., 2019; Wahis et al., 2021). Aside from OTR expression in PKCδ + inhibitory cells in CeL, recent reports suggest that a morphologically distinct subpopulation of astrocytes in CeL express OTR and these cells strongly mediate the anxiolytic and positive reinforcement effects of OT (Wahis et al., 2021). Consistent with this mechanism, OTR + cellular activation in CeL rescues adaptive avoidance behavior in rats deprived of inputs from the basolateral amygdala. In these animals, electrophysiological recordings showed an enhanced α-amino-3-hydroxy-5-methyl-4-isoxazolepropionic acid (AMPA)-dependent connectivity between the LA and CeL OTR + neurons but not OTR- neurons in high avoiders, i.e., animals that learned to avoid an imminent threat (Terburg et al., 2018). These findings highlight the role of OT within the centrolateral amygdala in modulating divergent defensive responses depending on the context and tone of threat/safety contingency (Ferretti et al., 2019).

### Oxytocin modulates stress/threat responses in the prefrontal cortex

Neural network activity in the rodent medial prefrontal cortex (mPFC) has been associated with the acquisition of threat responses and their extinction. Along the dorsoventral axis, mPFC is divided into two subregions crucial for threat responses – the prelimbic cortex (PL) and the infralimbic cortex (IL). While PL promotes the expression of freezing, the IL is involved in its extinction (Milad and Quirk, 2002; Burgos-Robles et al., 2007; Sotres-Bayon and Quirk, 2010; Sierra-Mercado et al., 2011). Thus, these two subregions of the mPFC are differentially recruited during the perception of threat versus safety (Knapska et al., 2012; Cain, 2019). Consistent with these findings, functionally distinct neurons in the BLA project to either PL and IL regions, and mediate freezing acquisition and extinction, respectively (Senn et al., 2014; Vogel et al., 2016). mPFC networks are also responsible for the acquisition, expression, and extinction of adaptive avoidance via GABAergic modulation (Capuzzo and Floresco, 2020; Diehl et al., 2020).

Oxytocin (OT) plays an important role in modulating prefrontal GABAergic control of mPFC activity. This function of OT in mPFC circuits is crucial for extinguishing defensive threat reactions (freezing) in rodents and humans (Eckstein et al., 2015, 2019; Sabihi et al., 2017; Triana-Del Río et al., 2019). Among cortical interneurons, OTR is primarily expressed in Som + cells in the rodent mPFC (Nakajima et al., 2014; Li et al., 2016), however, OTR expression varies by sex and brain state. For instance, OTR expression increases in lactating females compared to virgins or male rodents (Mitre et al., 2016). In mPFC, OTR modulates stress responses in a sex-dependent manner, mainly through its interaction with CRF stress-dependent signaling (Li et al., 2016), and potentially with sex hormones like estradiol (Gruene et al., 2015). Also with ng this finding, in a subregion-specific manner, OT signaling reduces anxiety-like behavior through its action in PL but not in IL (Sabihi et al., 2014). Complementarily, blocking OTR in PL is anxiogenic in lactating dams but not in virgins. This mechanism appears to be GABA-dependent, as administration of OT in the PL was accompanied by increased activation of GABA neurons in the same area (Sabihi et al., 2017). This finding supports the observation that OTR is expressed primarily by PL SOM interneurons, which is also consistent with the idea that this cell population is responsible for controlling the discrimination of affective states in rodents (Scheggia et al., 2020).

### Oxytocin modulates stress/threat responses in the bed nucleus of the stria terminalis

The bed nucleus of the stria terminalis (BNST) is evolutionarily and anatomically close to the CeL. Of the BNST cell types, type III consists mainly of OT-positive GABAergic neurons. In this area, OT modulates adaptive responses to threats and attenuates fearful reactions in animal models and human studies via a possible interaction with the CRF system (Janeček and Dabrowska, 2019; Mosley et al., 2021). Endogenous OT excites BNST interneurons and inhibits CeL-driven BNST output neurons by releasing GABA (Francesconi et al., 2021). The aforementioned neuromodulatory mechanism serves to enhance threat memory for a discrete cue or landmark (freezing behavior), enabling accurate and rapid adaptive responses to the threat, for example, in stress-induced social vigilance and adaptive avoidance (Martinon and Dabrowska, 2018; Steinman et al., 2019; Duque-Wilckens et al., 2020).

## Limbic forebrain-based oxytocin signaling network in social behaviors

In addition to stress/threat responses, OT also plays a vital role in modulating social behaviors in rodents. Animals engage in a variety of social behaviors including social interaction, pair bonding, sexual behaviors, maternal care, and aggression (Jurek and Neumann, 2018). Among these behaviors, appetitive social behaviors toward a conspecific are determined using assays to measure social approach/avoidance, which refers to the animal’s tendency to engage in social interaction often in preference to exploring a non-social object. Motivation to engage in social approach/avoidance can be assessed as socially conditioned place preference (CPP). Social memory/recognition involves the initial sensing of a conspecific through sensory modalities followed by memory formation and recall of a previously encountered conspecific. Lastly, social threat-related responses to a conspecific involve identifying, recognizing a threat, and responding appropriately. There is compelling evidence that OT signaling plays an important role in mediating multiple aspects of social behaviors – encompassing social investigation, social motivation, social memory, and social threat.

Global manipulation of the OT system provides insight into its modulatory influence on social behaviors. Systemic administration of OT or OTR agonist (OT-A) increases time spent in social investigation and augments social preference in a social CPP task (Ramos et al., 2015; Sobota et al., 2015; Zhang et al., 2015; Kohli et al., 2019; Duarte-Guterman et al., 2020). Of note, this effect of OT is both sex-specific and age-dependent (Zhang et al., 2015; Dannenhoffer et al., 2018; Duarte-Guterman et al., 2020). Similarly, administration of an OTR antagonist decreases time spent in social investigation compared to vehicle-treated animals (Lukas et al., 2011). In a compelling study, investigators tested social discrimination in OT knockout (KO), OTR KO, and partial forebrain OTR KO mouse strains (OTR Fb KO). The transgenic subjects were all male mice of the C57BL/6J strain and tested with female Balb/c, C57BL/6J, and SW strains. Interestingly, OT KO and OTR KO mice showed impaired social memory in some strains and not others, whereas OTR Fb/Fb mice showed impaired social memory in all strains. This finding implies that OT plays a role in social recognition across different strains, who may exhibit distinct social cues and release diverse pheromones (Macbeth et al., 2009). While these studies elucidate the effects of the global OT system on social behaviors, brain region-specific manipulations of OT in the limbic forebrain have begun to reveal fascinating circuit mechanisms of this neuromodulator (Figure 1B and Table 1).

### Oxytocin signaling through the paraventricular nucleus is essential for social behavior

Oxytocin (OT) signaling in the rodent hypothalamus has been strongly implicated in social behaviors. While OT is synthesized by multiple hypothalamic nuclei, the PVN OT network is particularly implicated in the social approach/avoidance and social recognition/memory. Activity in OT neurons in the PVN has been shown to increase during social interaction (Hung et al., 2017). In addition, OT mRNA abundance in PVN significantly decreased in mice that showed low levels of social interaction compared to mice that showed high levels of social interaction (Murakami et al., 2011). These findings demonstrate that OT activity in PVN is positively correlated with social behaviors. As previously discussed, chronic restraint stress leads to an increase in OT immunoreactive cells in the PVN. This effect of chronic stress is accompanied by an increase in social approach (Li et al., 2016). OT signaling within PVN is also associated with rewarding aspects of social investigation. For instance, in a social CPP test - mice that show social preference have higher OT gene expression in the PVN compared to controls (Liu et al., 2016). More direct evidence for the role of OT neurons in the PVN in social behavior comes from Resendez and colleagues (Resendez et al., 2020), who showed that chemogenetic activation of OT neurons in the mouse PVN enhances social investigation while chemogenetic inhibition of the same neurons abolishes social investigation. Two-photon calcium imaging of PVN OT neurons in behaving animals has further revealed that these neurons are activated by social stimuli and they differentially encode social and non-social stimuli (Resendez et al., 2020). Together, these findings indicate that OT signaling in the PVN affects the salience of social behaviors.

In addition, social stress increases the release of OT, following heightened PVN network activity. This leads to an increase in OT-OTR binding in networks such as the lateral septum, where OT modulates the expression and extinction of social-derived threat responses in lactating females, which appears to be augmented by sex hormones (Zoicas et al., 2014; Menon et al., 2018). Similarly, in the PVN of female rodents, increased OT release is correlated with high levels of maternal aggression (Bosch et al., 2005). Excessive or uncontrollable socially induced stress is associated with OT signaling impairments, which in turn correlate with high levels of anxiety-like behavior. Consistent with these studies, exogenous OT promotes resilience to social stress (Hale et al., 2021; Shi et al., 2021), primarily through the involvement of projections from PVN OT neurons to the prelimbic cortex (He et al., 2019).

### Oxytocin signaling in the amygdala modulates social recognition/memory

Electroencephalogram (EEG) studies have shown that chronic OT administration leads to a decrease in high-frequency bands in the amygdala concurrent with increased social interaction compared to vehicle control. This indicates that OT might reduce local circuit activity within the amygdala, as effects on long-distance connectivity would have altered lower frequency bands (Sobota et al., 2015). Magnocellular OT neurons in the PVN project to the CeL and CeM subnuclei of the amygdala (Sofroniew, 1983). Besides the central amygdala, OTRs are also expressed in the BLA and medial amygdala (MeA) (Eckstein et al., 2015). To investigate the odor-induced recruitment of brain regions, Arakawa and colleagues (Arakawa et al., 2010) conducted an odor investigation test where a rat was placed in a cage with bedding that had the odor of a conspecific. Following the test, the authors carried out immunohistochemistry for cFos, a marker of cellular activity. This study showed that conspecific odor increases IEG expression in several brain regions including the olfactory bulb, MeA, BNST, and PVN. The increase in IEG expression was accompanied by an increased level of OTRs in the MeA and PVN. Further, they showed that infusion of OTR antagonist in the MeA decreases odor investigation compared to vehicle control. These findings provided compelling evidence for OT signaling within MeA in integrating conspecific odor-induced social investigation.

Moreover, OTR mRNA levels are significantly lowered in MeA of mice that showed low levels of social interaction compared to mice that had high levels of social interaction (Murakami et al., 2011). Ferguson and colleagues (Ferguson et al., 2002) examined cFos expression in OT KO and wild-type mice during a social learning task and reported similar activation in the main and accessory olfactory bulbs, the piriform cortex, and the cortical amygdala. Interestingly, wild-type mice had more activation in MeA compared to KO mice. Further, the BNST and medial preoptic area, which receive direct input from MeA, failed to show an increase in cFos expression in OT KO mice. Additional evidence for the role of MeA in social recognition comes from a study in female rats where re local infusion of antisense oligonucleotides targeting OTR resulted in impaired social memory (Choleris et al., 2007). Acute administration of OT into the MeA before memory acquisition rescued social memory (Ferguson et al., 2000), implying that local OT and OTR signaling in MeA are both necessary and sufficient for social recognition. Similar findings were reported in another study where the infusion of OTR antagonist in MeA impaired social memory in adult but not juvenile male rats (Lukas et al., 2013).

Oxytocin (OT)-induced protein synthesis is thought to be important for the consolidation of social memories. A study using social discrimination tasks found that blocking protein synthesis before memory acquisition blocked long-term social memory in rats. *In vitro*, exogenous administration of OT has been shown to enhance theta burst stimulation (TBS)-induced LTD in the anterior olfactory bulb (AOB)-MeA pathway. *In vivo*, TBS administered to the AOB before memory acquisition leads to social recognition deficits (Gur et al., 2014). Together, these findings show that MeA plays a crucial role in social memory and that OT modulates synaptic plasticity that is associated with social memory. More recent studies show that OT signaling in another amygdala subnucleus, BLA, affects social behavior. Social recognition was impaired in female mice that underwent early life stress, which coincided with an increase in dendritic complexity as well as an increase in OTR density within the BLA (Wei et al., 2015). Further, optogenetic stimulation of OTR-expressing PFC neurons that project to the BLA eliminated social recognition (Tan et al., 2019). Though these early studies are promising, more investigation into OT signaling in the BLA is needed to understand how this brain area is contributing to social approach and social memory.

### Oxytocin signaling in the prefrontal cortex modulates social interaction and social threat

Oxytocin (OT) signaling in mPFC has been shown to modulate social behaviors such as social recognition and social threat. A recent study showed that a subset of glutamatergic neurons in mPFC projecting to BLA express OTRs, separately from cortical interneurons. This study demonstrated that optogenetic stimulation of the mPFC-BLA pathway impairs social recognition (Tan et al., 2019). Another study demonstrated that social isolation for over a week leads to increased subsequent social interaction, which is accompanied by increased excitatory neurotransmission from the ventral tegmental area (VTA) afferent to the mPFC. Chemogenetic inhibition of OT neurons in PVN abolishes social isolation-dependent modifications of synaptic efficacy and behavior, indicating a crucial role of OT signaling across VTA and mPFC for motivated social behavior (Musardo et al., 2022). Additionally, gene deletion of an inhibitory subunit of NMDA receptor (GluN3A KO) results in a significant reduction in OTR expression within mPFC and impaired social interaction in KO mice, which is rescued with exogenous OT administration (Lee et al., 2018).

Oxytocin (OT) signaling in mPFC also plays a key role in integrating social and threat-related information for optimal behavior outcomes. In a phenomenon known as social buffering, stress and fear responses are attenuated by acute or chronic social exposure. Jang and colleagues showed that paired rats exhibit reduced threat responses in a passive avoidance task and that OT infusion in the prelimbic cortex within mPFC of social-deprived rats results in a similar reduction in threat responses (Jang et al., 2022). Interestingly, social buffering of stress/threat response requires nascent protein synthesis in mPFC (Jang et al., 2022). Hence endogenous oxytocin seems to be fundamental for mediating the buffering effects of social interactions to diminish threat reactions like freezing (Ferrer-Pérez et al., 2020; van den Burg and Hegoburu, 2020).

## Oxytocin modulation of the mesolimbic dopamine system in socio-emotional behaviors

The mesolimbic dopamine system connects the ventral tegmental area (VTA) with the ventral striatum, which includes the nucleus accumbens (NAc) and the olfactory tubercle. VTA in the telencephalon is populated by dopaminergic neurons and plays a significant role in reward, motivation, cognition, and aversion. On the other hand, the ventral striatum is populated by medium spiny neurons that express receptors for dopamine released by afferents from VTA. OTR is highly expressed in the mesolimbic dopamine circuit, including in the medial and lateral subdivisions of VTA. At a cellular level, OTRs are expressed in glutamatergic and dopaminergic neurons whose fibers project to the NAc, prefrontal cortex, and amygdala (Peris et al., 2017).

Direct activation of PVN-VTA projecting OT neurons leads to an increase in social investigation. On the other hand, inhibiting PVN-VTA OT neurons leads to abolished social preference in a social CPP test, indicating that this projection is required for social reward (Hung et al., 2017). Whole-cell recordings in the midbrain dopamine system have revealed that bath application of OT increases the firing rate of dopamine neurons in VTA (Xiao et al., 2017). Studies carried out on OT signaling in the striatum have similarly elucidated an important role of OT in mediating social behaviors. For example, Zhang and colleagues (Zhang et al., 2015) investigated the effects of OT infusion in the striatum and found that both males and females increase social interaction following the treatment. The same study also performed proteomics of striatum and found that protein levels of calcineurin and GAD67 were significantly altered upon OT administration. Specifically, calcineurin levels increased whereas GAD67 levels decreased in the striatum, indicating a possible role of OT in modulating excitatory-inhibitory balance in the striatum. Moreover, OT directly influences dopamine release in the mesolimbic circuit. Intraperitoneal injection of OT in rats leads to an increase in persistent dopamine release in the NAc (Kohli et al., 2019). Bath application of OT caused long-term depression (LTD) in medium spiny neurons in the NAc whereas application of an OTR antagonist occluded the LTD. Further investigation revealed that this synaptic plasticity was caused by a decrease in the probability of presynaptic neurotransmitter release. At the behavior level, infusion of OTR antagonist within the NAc of male mice prevented the preference for a socially conditioned context in a social CPP test (Dölen et al., 2013). The effects of OT on the mesolimbic dopamine system appear to be age-dependent. Administration of an OTR antagonist into the NAc, but not BLA, decreases time spent in the investigation of the novel conspecific, which shows that OT in the NAc is essential for motivating social behavior (Smith et al., 2017). Therefore, OT acts on the mesolimbic dopamine system to modulate reward-processing related to the social approach.

In addition to its influence on social behaviors, the mesolimbic dopamine system is recruited during contextual threat responses and is associated with neural circuit function in brain areas where OTRs are also expressed – such as the amygdala, prefrontal cortex, VTA, and striatum. There is evidence that dopaminergic neurons increase their spiking activity during the extinction of freezing responses and prevent the renewal of such responses (Bouchet et al., 2018). Dopamine also gates the associative learning of fear to switch from danger to safety conditioning (Groessl et al., 2018; Luo et al., 2018). Concerning stress-response, subchronic social isolation stress leads to OT-mediated strengthening of excitatory neurotransmission from VTA to mPFC (Musardo et al., 2022). Thus, the interactions between the mesolimbic dopamine system and OT signaling seem to be important not only for social behaviors but also for mediating stress/threat responses.

## Oxytocin dysfunction and therapeutic potential in neuropsychiatric disorders

Oxytocin signaling has been implicated in a wide range of neuropsychiatric disorders, due to its crucial role in emotional and social behaviors. Among these disorders, autism spectrum disorders (ASD) are characterized by repeated or restricted interests and deficits in social interaction and social communication. Interestingly, the same brain regions that have been associated with stress/threat and social responses in rodents show abnormal activity in ASD patients (Dichter, 2012). In rodent models of ASD, OT administration has been shown to ameliorate social deficits (Peñagarikano et al., 2015; Wang et al., 2018; Hörnberg et al., 2020; Cherepanov et al., 2021). In clinical studies, intranasal OT has been used to treat ASD. So far, results have been mixed; some studies find that intranasal OT improves social deficits (Huang et al., 2021), while others report no effects on social deficits (Yamasue et al., 2020; Sikich et al., 2021). Some studies attribute the different responses to OT to differences in individual variability (Kosaka et al., 2016; Parker et al., 2017), though these results have also been challenged (Sikich et al., 2021). Further studies demonstrating the long-lasting effects of stress attenuation by OT are warranted, especially for social threats. This would provide the neurobiological basis for treatment options for social dysfunctions using exogenous OT (Neumann and Slattery, 2016).

Studies in animals strongly support the role of the OT-OTR system in the modulation of traumatic or threat memories (Triana-Del Río et al., 2019). In clinical studies, this system has been investigated in post-traumatic stress disorder (PTSD), an emotional disorder characterized by over-consolidation of traumatic/threat memories, generalization of fear, hyperactivity of the amygdala and attempts to avoid trauma-related cues. Findings from studies involving OT as a therapeutic strategy for PTSD show that an acute dose of intranasal OT increases neural responses to social reward (Nawijn et al., 2017), whereas repeated OT administration reduced symptoms of PTSD, which is correlated with decreased amygdala reactivity to fearful faces, and the attenuation of amygdala-PFC functional connectivity (Frijling, 2017). Intranasal administration of OT has been shown to trigger behavioral and neuronal responses related to threat memory processing in PTSD patients, suggesting that, similar to animal models, OT can amplify the acquisition and consolidation of threat/intrusive memories, on a comparable process of increasing the signal to noise ratio for relevant threat stimuli (Owen et al., 2013; Terburg et al., 2018). In these studies, the effects of exogenous OT are influenced by biological covariates, such as salivary cortisol, heart rate variability, sex, and PTSD polygenic risk scores (Schultebraucks et al., 2022). On the other hand, another group demonstrated that OT administration early after trauma did not attenuate PTSD symptoms in all trauma-exposed participants with acute distress; however, participants with high acute PTSD symptom severity did show beneficial effects of OT (van Zuiden et al., 2017). Following these efforts, Carmassi and colleagues (Carmassi et al., 2021) showed that baseline plasma OT levels are decreased in PTSD patients of both sexes compared to healthy controls. However, in another study, the change in peripheral OT levels did not differ by treatment condition and did not correspond to chana get in PTSD symptoms (Sippel et al., 2021).

Converging translational studies indicate that intranasal administration of OT reaches the brain to trigger physiological effects (Quintana et al., 2019, 2021; Winterton et al., 2021), correlated with a general OT-concentration increment in salivary, plasma, and cerebrospinal fluid samples (Striepens et al., 2013). However, it is still unknown if these effects reflect amplified endogenous OT release upon intranasal OT administration (as a result of positive feedback mechanisms) or the exogenously administered compound (Quintana et al., 2019, 2021). In clinical studies using OT, the statistical power is limited, thus, more systematization, larger sample sizes, and diversity in the sample populations are required, making emphasis on the exact duration of OT administration, its concentration, the dose-response effects, and the stress reactivity of the participants (Quintana et al., 2021; Winterton et al., 2021). Therefore, converging literature support the critical role of OT in social and emotional behaviors in both rodents and humans, but the therapeutic potential of OT on human neuropsychiatric disorders remains to be fully characterized.

## Conclusion

While not comprehensive, we have highlighted here the studies that provide compelling evidence on how OT signaling in the limbic network modulates social and stress/threat-related behaviors. At the intersection of social behaviors and stress/threat response is social threat response when the conspecific partner is perceived as a threat. OT exerts an essential role in increasing the signal-to-noise ratio of sensory signals within a social context (Owen et al., 2013; Marlin et al., 2015; Tirko et al., 2018); this effect is potentiated when the social context becomes stressful or threatening context. OT provides resilience against social stress and socially derived threat responses. There are two prominent modulations of the OT system related to social threat, and both of them seem to relate to the increased OT release as a survival signal against the stress/threat context. First, OT is released during social buffering of acute stress responses. For example, OT signaling in the prelimbic cortex reduces freezing expression induced by acute social exposure (Jang et al., 2022), a phenomenon that has been observed in the neighboring anterior cingulate cortex (Burkett et al., 2016), and PVN (Smith and Wang, 2014), hence endogenous oxytocin seems to be fundamental for mediating the buffering effects of social interactions to diminish threat reactions like freezing (Ferrer-Pérez et al., 2020; van den Burg and Hegoburu, 2020). Secondly, social stress also increases the release of OT, as mentioned before for PVN network activity. This increases OTR binding in networks like the lateral septum, where OT modulates the expression and extinction of socially derived threat responses in lactating females, which appears to be tempered by sex hormones (Zoicas et al., 2014; Menon et al., 2018). In this brain area, OT signaling promotes rather exacerbated threat responses (freezing) and aggression responses to social threats (Guzmán et al., 2013; Meyer et al., 2020), similarly, in the PVN of rodent females, increased OT release is correlated to high levels of maternal aggression (Bosch et al., 2005). Excessive or uncontrollable socially induced stress is associated with OT signaling impairments, which are also correlated to high levels of anxiety-like behavior. This is described as the nucleus accumbens (Hou et al., 2020) or the mPFC (Shi et al., 2021). To counterbalance this effect, exogenous oxytocin promotes resilience to social stress (Hale et al., 2021; Shi et al., 2021), primarily through the involvement of projections from PVN OT neurons to PL (He et al., 2019). Therefore, coordinated regulation of neural circuits across the limbic forebrain is necessary for OT-mediated behavioral and physiological responses to social, stress, and threat-related stimuli when presented in isolation or together.

The crucial challenge in oxytocin research is determining how context-dependent intracellular signaling responses elicit a particular behavioral or physiological response. OTR-induced cellular responses and the signaling mechanisms in behaviorally relevant neural circuits may provide a better understanding of these effects. Behavioral neuroscience will significantly benefit from this knowledge. This knowledge of signaling cascades and secondary messengers will further aid in treatments using oxytocin and its analogs which will facilitate the design of better therapeutic interventions for neuropsychiatric disorders involving oxytocin.

## Author contributions

RT-D, SR, JG, and PS: manuscript writing and proofreading. JL and EK: manuscript proofreading. All authors contributed to the article and approved the submitted version.
